# Chemical Composition, Antioxidant and Enzyme Inhibitory Properties of Different Extracts Obtained from Spent Coffee Ground and Coffee Silverskin

**DOI:** 10.3390/foods9060713

**Published:** 2020-06-02

**Authors:** Gokhan Zengin, Kouadio Ibrahime Sinan, Mohamad Fawzi Mahomoodally, Simone Angeloni, Ahmed M. Mustafa, Sauro Vittori, Filippo Maggi, Giovanni Caprioli

**Affiliations:** 1 Department of Biology, Science Faculty, Selcuk University, Campus, Konya, 42130 Konya, Turkey; gokhanzengin@selcuk.edu.tr (G.Z.); sinankouadio@gmail.com (K.I.S.); 2 Institute of Research and Development, Duy Tan University, Da Nang 550000, Vietnam; mohamadfawzimahomoodally@duytan.edu.vn or; 3 Department of Health Sciences, Faculty of Science, University of Mauritius, Réduit 80837, Mauritius; 4 School of Pharmacy, University of Camerino, Via Sant’Agostino 1, 62032 Camerino, Italy; simone.angeloni@unicam.it (S.A.); ahmed.mustafa@unicam.it (A.M.M.); sauro.vittori@unicam.it (S.V.); giovanni.caprioli@unicam.it (G.C.); 5 International Hub for Coffee Research and Innovation, Via E. Betti 1, 62020 Belforte del Chienti, Italy

**Keywords:** antioxidants, polyphenols, silverskin, spent coffee ground, enzyme inhibitory properties, HPLC

## Abstract

In a world where an eco-friendlier approach is becoming more and more necessary, it is essential to reduce waste production and to reuse residues of the company’s supply chain. Coffee silverskin (CS) and spent coffee ground (SCG), two by-products of coffee production, are important sources of bioactive compounds and, for this, some authors have proposed their reuse in the nutraceutical, food, and cosmetic sector. However, their potential enzyme inhibitory properties have been poorly investigated. Hence, the objective of the current work was to study the enzymatic inhibitory activities against acetylcholinesterase, butyrylcholinesterase, α-amylase, α-glucosidase, and tyrosinase of different extracts of CS and SCG. Before these in vitro bioassays, the phytochemical composition of each extract was investigated via colorimetric assays and HPLC-MS/MS analysis. In addition, the antioxidant activities were evaluated by different chemical approaches. SCG extracts contained a higher content of bioactive compounds, notably the SCG EtOH:H_2_O extract was the richest in caffeine and possessed the highest antioxidant activities. The hydroalcoholic and methanolic extracts were shown to be the most active against all tested enzymes, while the water extracts displayed lower activity. Our results showed a weak correlation between bioactive compounds and enzyme inhibitory effects, proving inhibitory activities likely due to non-phenolic molecules such as alkaloids and terpenoids. Obtained findings could be a starting point to develop novel nutraceuticals from CS and SCG.

## 1. Introduction

Coffee is one of the most consumed beverages in the world and, in addition, coffee beans are considered an important agricultural product of the international trade [1]. According to the latest International Coffee Organization (ICO) statistics, global coffee consumption for the year 2019–2020 has reached 169 million of 60-kg bags, with Europe accounting for about one third of the global consumption [2]. Therefore, to satisfy the enormous product demand, coffee companies must process a huge quantity of raw material, causing a release of a notable amount of solid and liquid residues, since about 90% in weight of coffee berries is discarded during manufacturing as agricultural waste and by-products [3]. Because of this, several authors have proposed the possible recycling of different coffee residues to be reused in various industrial sectors, and at the same time, reducing the quantity ending up in landfills [4,5,6,7,8]. Among them, coffee silverskin (CS) and spent coffee ground (SCG) are two important by-products of coffee processing; the first one, a thin tegument which covers the coffee seeds, is released during roasting, while the second one is the residue obtained in large part from the soluble coffee company and brewing process [9]. Some authors have proposed the use of both by-products for different uses which affect various fields; for instance, some researchers have suggested to employ them as adsorbent material for removing potential toxic metals [4], while some studies have dealt with the possibility to recover bioactive compounds from these two co-products and employing them in the food, pharmaceutical and cosmetic industry [3,10,11]. In this context, interesting works took advantages of their high content of dietary fibers, phenolic compounds, chlorogenic acids and caffeine for the formulation of fortified foods [12,13]. In fact, SCG and CS contain a variegated phytochemical profile characterized by diverse biological activities, e.g., antioxidant and antimicrobial [7,14,15,16]. However, to the best of our knowledge, a comprehensive chemical characterization and an extensive antioxidant activities evaluation for CS and SCG extracts is lacking. For instance, some authors have studied the antioxidant activities by ferric reducing antioxidant power (FRAP) assay of SCG extracts [14], while other researchers have performed the free radical 2,2-diphenyl-1-picrylhydrazyl method (DPPH) and oxygen radical absorbance capacity (ORAC) assay of a purified SCG extract [15]. DPPH and FRAP were performed in another work for different CS extracts [16]. In fact, the study of antioxidant activities should include, if in vivo investigation is not feasible, at the very least, methods based on distinct mechanisms of action, i.e., single electron transfer, transition metal chelating ability, and hydrogen atom transfer [17]. In addition, to the best of our knowledge, there is a dearth of investigations on the enzyme inhibitory properties of CS and SCG extracts, although coffee and most likely its by-products contain some potential enzyme inhibitors such as caffeine, chlorogenic acids and some unconjugated phenolic acids and flavonoids [18,19,20,21]. The enzyme inhibitors have been used for the treatment of several diseases since several years and therefore they are becoming very attractive targets for drug discovery and development [22]. Hence, account taken of all the above, the objective of the present research was to characterize the phytochemical profile of different extracts of CS and SCG and to study their antioxidant and enzyme inhibitory properties in the perspective of their application in the pharmaceutical and nutraceutical industry. For this purpose, HPLC-MS/MS studies were performed to quantify 25 bioactive compounds including alkaloids, phenolic compounds, and flavonoids in addition to colorimetric assays such as total phenolic content (TPC) and total flavonoid content (TFC). Then, different tests including diverse mechanisms of action such as metal chelating, phosphomolybdenum, ferric reducing power (FRAP), cupric reducing antioxidant capacity (CUPRAC), 2,2′-azino-bis (3-ethylbenzothiazoline-6-sulphonic acid) (ABTS), and 2,2-diphenyl-1-picrylhydrazyl (DPPH) activities, were performed to evaluate the antioxidant properties of each extract. Moreover, the enzymatic inhibitory activities against acetylcholinesterase (AChE), butyrylcholinesterase (BChE), α-amylase, α-glucosidase, and tyrosinase were assessed using standard in vitro bioassays. Finally, statistical analysis was applied to study (a) the correlation between the different extracts produced from SCG and CS based on their chemical composition (principal component analysis, PCA), (b) the relationship between total bioactive compounds (TPC and TFC) and tested biological properties (Pearson’s and Spearman correlation coefficient), (c) the most pharmaceutically efficient by-product and the biological activities responsible for sample discrimination (supervised partial least squares discriminant analysis, PLS-DA), and (d) the effects of extraction solvents on biological activities of each extract (normality tests and univariate analysis).

## 2. Materials and Methods

### 2.1. Chemicals and Reagents

Cyanidin-3-glucoside chloride, delphinidin-3,5-diglucoside chloride, kaempferol-3-glucoside were purchased from PhytoLab (Vestenbergsgreuth, Germany). The other 22 analytical standards of the monitored bioactive compounds were supplied by Sigma-Aldrich (Milan, Italy). Individual stock solutions of each analyte, at a concentration of 1000 µg mL^−1^, were prepared by dissolving pure standard compounds in HPLC-grade methanol and storing them in glass stoppered bottles at 4 °C. Afterwards, standard working solutions at various concentrations were daily prepared by appropriate dilution of the stock solution with methanol. HPLC-grade formic acid 99–100% was purchased from Merck (Darmstadt, Germany) while HPLC-grade methanol was supplied by Sigma-Aldrich. Deionized water was obtained from a Milli-Q reagent water system (Bedford, MA, USA). All other solvents and chemicals were analytical grade. Before HPLC analysis, all solvents and solutions were filtered through a 0.2 µm polyamide filter from Sartorius Stedim (Goettingen, Germany) and all samples were filtered with Phenex™ RC 4 mm 0.2 µm syringeless filter (Phenomenex, Castel Maggiore, Italy).

### 2.2. Coffee Silverskin and Spent Coffee Ground Sample

Green beans of 100% *Coffea arabica* L., Ethiopian origin, was supplied from Simonelli Group S.p.A. (Belforte del Chienti, Italy). Coffee silverskin (CS) and roasted beans were collected by roasting green beans using an Ikawa coffee roaster (Ikawa Ltd., London, UK). The roasting process was performed at a maximum temperature of 190 °C for a total roasting time of 8 min. The resulting CS were stored at 4 °C until use. Roasted beans were milled by Mythos 1 coffee grinder (Simonelli Group S.p.A.) and spent coffee ground (SCG) was obtained by brewing roast and ground (R&G) coffee with a semiautomatic espresso coffee machine (VA388 Black Eagle, Victoria Arduino, Simonelli Group S.p.A.) following these conditions: 7 ± 0.05 g of R&G coffee per cup, 25 ± 1 s of extraction, water pressure and temperature 9 bar and 92.0 °C, respectively, and 25 ± 2 g in cup. After a series of espresso coffee replicates, SCG was collected and oven-dried at 50 °C until constant weight (48 h). Dried sample were stored at 4°C until use.

### 2.3. Preparation of CS and SCG Extracts

Just before the extraction, CS was immersed in liquid nitrogen and milled by Ariete Blendy 570 grinder (Florence, Italy). Eight different extracts of CS and SCG were prepared following previous extraction procedures [7,23] with some adjustments. The bioactive compounds in 10 g of CS powder and SCG were extracted with 50 mL of solvent using a FALC ultrasonic bath (FALC, Treviglio, Italy) at a frequency of 40 kHz for 120 min at 20 °C. After the extraction, the sample was filtrated with a filter paper and the obtained extract was collected, lyophilized and stored in darkness at a temperature of −20 °C until analysis. For the current work, four different solvents were selected due to their performances in terms of recovery and extraction yield [7]. The selected solvents were H_2_O, MeOH, MeOH:H_2_O (50:50), and EtOH:H_2_O (70:30) and from now on, the resulting extracts are simply referred to as CS H_2_O, CS MeOH, CS MeOH:H_2_O, and CS EtOH:H_2_O for CS and SCG H_2_O, SCG MeOH, SCG MeOH:H_2_O, and SCG EtOH:H_2_O for SCG. Before analyses, 5 mg of lyophilized extract were dissolved in 5 mL of MeOH (1 mg mL^−1^) sonicated for 10 min. For HPLC analyses, an aliquot of the solution was filtrated with a 0.2 μm pore size filter and then injected into HPLC-MS/MS.

### 2.4. Total Phenolic and Flavonoid Content

Spectrophotometric methods were conducted to determine the total phenolic and flavonoid contents of CS and SCG extracts and detailed methods were described in our previous paper [24]. Standards, namely gallic acid (GAE) for phenolics and rutin (RE) for flavonoids, were used to explain the results.

### 2.5. Bioactive Compound Quantification by HPLC-MS/MS

Five mg of lyophylized extract were dissolved in 5 mL of HPLC-grade MeOH (1 mg mL^−1^) sonicated for 10 min, and an aliquot of the solution was filtered using a 0.2 µm syringeless filter and then injected into the HPLC-MS/MS system. The quantitation of 25 bioactive compounds was performed by following developed procedures with some modifications [7,23]. Briefly, the analytical studies were carried out by using a 1290 Infinity series high performance liquid chromatograph (Agilent, Santa Clara, CA, USA) and a 6420 Triple Quadrupole (Agilent) equipped with an electrospray ionization (ESI) source operating in negative and positive ionization mode. A Kinetex PFP analytical column, 100 mm × 2.1 mm i.d., particle size 2.6 µm (Phenomenex, Torrance, CA, USA) was used for analyte separation. The mobile phase was a mixture of water (A) and methanol (B) both containing 0.1% of formic acid. The elution was performed in gradient mode at a flow rate of 0.2 mL/min. The composition of the mobile phase varied as follows: 0–2 min, isocratic condition, 20% B; 2–15 min, 80% B; 15–18 min, isocratic condition, 80% B; 18–23 min, 100% B; 23–35 min, 20% B. The injection volume was 2 µL and the temperature of the drying gas in the ionization source was 350 °C. The gas flow was 10 L min^−1^, the nebulizer pressure was 172.369 kPa and the capillary voltage was 4000 V. Detection was performed in the Dynamic “multiple reaction monitoring” (Dynamic-MRM) mode and the Dynamic-MRM peak areas were integrated for quantification. The most abundant product ion was used for quantitation, and the others to confirm the analyte. The selected ion transitions and the mass spectrometer parameters including the specific time window for each compound (delta retention time) are reported in Table S1.

### 2.6. Determination of Antioxidant and Enzyme Inhibitory Effects

To detect antioxidant properties, we used several chemical assays including different mechanisms, namely radical scavenging, reducing power and metal chelating. Trolox (TE) and ethylenediaminetetraacetic acid (EDTA) were used as standard antioxidant compounds. Obtained results were expressed as equivalents of these compounds [25]. To detect inhibitory effects on enzymes, we used colorimetric enzyme inhibition assays including tyrosinase, α-glucosidase, α-amylase and cholinesterases. The standard inhibitors galatamine, kojic acid and acarbose were used as positive controls. The details of experiments are given in Supplemental Materials.

### 2.7. Statistical Analysis

In order to study the correlation between the different extracts produced from SCG and CS based on their chemical composition and to identify the main factors influencing the samples variability, a data matrix with 22 variables × 8 samples was created and subjected to principal component analysis (PCA) using the STATISTICA v. 7.1. software (Stat Soft Italia S.r.l., Vigonza, Italy). Before proceeding, data were normalized: missing data were substituted by the value 0.001 whereas compounds without variance were not included. Eigenvalues were determined using a covariance matrix and score and loading plots, representing extract samples and chemical compounds, were obtained.

Outcomes of replicate readings were aggregated and given as mean ± SD standard deviation. Shapiro–Wilk and Anderson–Darling tests were achieved to check the normal assumption of data distributions. According to result of normality test, Kruskal–Wallis or one-way analysis of variance were performed to assess the significant differences among samples (Tables S2 and S3). Difference was considered significant at the 5% confidence level (Dunn or Turkey’s post-hoc). The relationship between total bioactive compounds (TPC and TFC) and studied biological activities was estimated calculating Pearson’s and Spearman correlation coefficient.

Thereafter, the dataset was submitted to supervised partial least squares discriminant analysis in an attempt to discriminate coffee residues. Model performance was recorded by estimating R^2^Y (goodness-of-fit), Q^2^Y (goodness-of-prediction), and AUC (area under curve) value. Variable importance in projection was used to determine the importance of each biological activity in coffee by-products separation. Biological activities with VIP (variable importance of projection) score > 1 were considered to have the highest discrimination potential (VIP score > 1) and Wilcoxon test was achieved to characterize coffee by-products taking into account the identified discriminant biological activities. Finally, dotplot was achieved following normality checking and univariate analysis (Kruskal–Wallis or one-way ANOVA) respectively, to view the effect of extraction solvents on biological activities of each extract. R v 3.6.1 statistical software (R Development Core Team, Vienna, Austria) was employed for the analysis.

## 3. Results and Discussion

### 3.1. Phytochemical Profile

#### 3.1.1. Total Phenolic and Flavonoid Content

Table 1 depicts the total amount of phenolics and flavonoids in SCG and CS extracts.

The total phenolic content in the tested extracts varied from 20.49 to 93.55 mg GAE g^−1^ extract. The total amount of phenolics in the SCG extract was higher than that of CS extracts; notably the SCG extracts can be ranked in the following order EtOH:H_2_O > MeOH:H_2_O > MeOH > H_2_O. In both by-product samples, the total quantity of phenolics was influenced by the solvent used for extraction. Among all the tested extracts, water was the least efficient solvent. These findings are consistent with earlier studies, which reported the lowest level of phenolics in water extracts from coffee or coffee by-products [7,26]. Regarding the total flavonoid contents, the order was similar to that of phenolics and the highest level of total flavonoid was determined in the SCG EtOH:H_2_O. The SCG extracts were richer than CS ones in terms of total quantities of phenolics and flavonoids. Several authors have reported different levels of total phenolic and flavonoids in coffee and its by-products and this was consistent with our results [9,27,28]. In recent years, the colorimetric methods for determination of phytochemicals are no longer considered as valid for obtaining accurate insights into the composition. For example, Folin-Ciocalteu reagent does not only react with phenolics and thus the obtained results might not be fully representative for phenolic levels in the extracts [29,30]. In this sense, obtained results from the colorimetric methods need to be verified by advanced chromatographic techniques such as HPLC-MS/MS and NMR amongst others. This is the reason why 25 bioactive compounds in SCG and CS extracts, including alkaloids, chlorogenic acids, unconjugated phenolic acids and flavonoids, have been analyzed using HPLC-MS/MS system.

#### 3.1.2. Content of 25 Bioactive Compounds in CS and SCG Extracts

Before analysis of different extracts, the analytical method was validated by studying linearity, sensitivity and precision. The linearity was evaluated by injecting seven different concentrations (0.001–10 µg mL^−1^) of standard mixtures of 25 analytes and then the calibration curves were plotted and the respective coefficients of correlation (*R*^2^) calculated. The current method showed satisfactory linearity for all target molecules since *R*^2^ ranged from 0.9945 to 1.0000. Limit of detection (LOD) and limit of quantification (LOQ) were calculated by injecting gradually lower concentrations of the standard mixture of 25 analytes and measuring signal-to-noise ratio (SNR). LOD was the concentration of the standard compound with SNR = 3, while LOQ with SNR = 10. The LODs obtained ranged from 0.0005 to 0.03 µg mL^−1^, while the LOQs were between 0.002 and 0.1 µg mL^−1^. Similar results have been reported in a previous work [7]. The precision of the method was measured by injecting three replicates of five different concentrations of standard mixtures of analytes over the course of three days. Relative standard deviation (% RSD) was used to express the run-to-run and day-to-day precision and the first ranged from 0.6 to 5.8%, while the second was between 3.3 and 11.4% for all the analytes.

Table 2 shows the content (expressed as µg g^−1^ of dry weight extract) of 25 bioactive compounds in the tested SCG and CS extracts. The total concentration of bioactive compounds varied from 35,867.67 to 68,698.72 µg g^−1^. In SCG, the highest concentration was found in EtOH:H_2_O extract (68,698.72 ± 5012.36 µg g^−1^), followed by MeOH:H_2_O (60,520.82 ± 4536.41 µg g^−1^), H_2_O (52,768.88 ± 3658.54 µg g^−1^) and MeOH (47,191.31 ± 3058.21 µg g^−1^) extracts. Similar behavior emerged in CS extracts since the highest content of total analytes was obtained in EtOH:H_2_O extract (50,428.89 ± 4023.74 µg g^−1^), followed by MeOH:H_2_O (47,702.54 ± 3987.36 µg g^−1^), H_2_O (36,601.49 ± 3025.45 µg g^−1^), and MeOH (35,867.67 ± 2898.32 µg g^−1^) extracts. In agreement with colorimetric methods, the highest content of analytes was found in SCG. It was influenced by the type of employed solvents and a mixture of EtOH:H_2_O was shown to be the most efficient in terms of bioactive compound contents, as it has been reported also in other works [7,23]. Among 25 monitored molecules, 21 analytes were over their LOQs in all SCG extracts, while 20 of them were quantified in almost all CS extracts. The analyte present at the highest concentration was caffeine which varied from 32,838.17 to 54,440.27 µg g^−1^ in SCG and from 27,365.14 to 41,877.13 µg g^−1^ in CS. Similar levels of caffeine were reported from Regazzoni et al. [26], who used an HPLC-UV system for the quantitative analysis. The extracts were good sources of chlorogenic acids as well since they occurred from 7605.10 to 14,104.31 µg g^−1^ in SCG and from 4264.56 to 8912.57 µg g^−1^ in CS. Among these conjugated phenolic compounds, 5-caffeoylquinic acid was the most abundant, followed by 3-caffeoylquinic acid, as shown in Table 2. Our analyses provided also an extensive characterization of phenolic acids; all seven monitored phenolic acids such as gallic, vanillic, caffeic, syringic, *p*-coumaric, ferulic and *trans*-cinnamic acid, occurred in the coffee extracts and vanillic (86.61–140.38 µg g^−1^), caffeic (45.32–204.95 µg g^−1^) and ferulic acid (38.69–145.32 µg g^−1^) were the most abundant ones. In addition, Monente et al. [31], who used a filter coffeemaker for SCG extract production, reported that caffeic and ferulic acid were the most abundant phenolic acids as determined by HPLC-DAD. In addition, different classes of flavonoids, i.e., flavonols (kaempferol-3-glucoside, quercetin, quercitrin, hyperoside, and rutin), flavan-3-ols ((+)-catechin and (-)-epicatechin), anthocyanidins (cyanidin-3-glucoside and delphinidin-3,5-diglucoside), and flavanones (naringin), an alkaloid (quinine), a xanthone (isogentisin), and other bioactive compounds such as resveratrol and shikimic acid, were monitored in the present work. Among flavonoids, rutin (1.25–10.65 µg g^−1^) and quercetin (3.06–3.76 µg g^−1^) were the most abundant ones in both by-product extracts, while quercitrin (0.12–0.83 µg g^−1^), kaempferol-3-glucoside (0.36–1.89 µg g^−1^), hyperoside (0.32–0.87 µg g^−1^), (+)-catechin (0.85–1.56 µg g^−1^), cyanidin-3-glucoside (0.93–1.89 µg g^−1^), and naringin (0.31–0.52 µg g^−1^) occurred at lower concentrations.

Interesting, (-)-epicatechin was found only in CS MeOH (123.65 ± 10.63 µg g^−1^) and CS MeOH:H_2_O (102.98 ± 8.65 µg g^−1^) extracts and this could partially explain the high levels of total flavonoid contents found in these two extracts (Table 1). Quinine (0.68–3.65 µg g^−1^), an alkaloid first isolated from the bark of the *Cinchona* tree and known as a potent antimalarial agent [32], and isogentisin (0.95–2.54 µg g^−1^), an important xanthone of the Gentian plant [33], were both found in the eight extracts.

**Figure 1:**
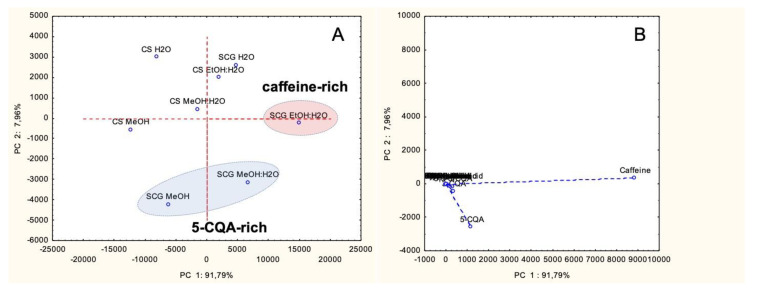
(**A**) Score plot generated by the principal component analysis (PCA) for the main variation in chemical constituents among extracts of silverskin (CS) and spent ground coffee (SCG). (**B**): The PCA loading plot for the chemical constituents representing 91.79% of the variation in the first principal component and 7.96% in the second principal component.

The PCA of the chemical compositions determined in the extracts from SCG and CS allowed to detect three main clusters. The PCA biplot is reported in Figure 1 and represents 99.75% of the total variance in the data matrix.

The variability was almost all generated along the first principal component (91.79%) and was given mostly by the content of caffeine (eigenvectors: 8830; 357) and, to a minor extent, 5-CQA (eigenvectors: 1159; −2559). Significantly lower was the influence of the second principal component on the total variability (7.96%) and was determined mostly by the variance of 5-CQA. On this basis, samples grouped in three different clusters, with extracts obtained from SCG using organic solvents being richer in caffeine (EtOH:H_2_O) and 5-CQA (MeOH and MeOH:H_2_O), whereas those from CS and the aqueous extract from SCG being poorer in these constituents. Notably, the EtOH:H_2_O and MeOH:H_2_O obtained from SCG revealed to have the strongest antioxidant capacity (see subsection below described, ‘3.2 Antioxidant capacity’) and this may be related to the higher content of bioactive caffeine and 5-CQA.

### 3.2. Antioxidant Capacity

To evaluate the antioxidant properties of SCG and CS extracts, we performed several in vitro assays. The obtained findings are summarized in Table 3.

**Figure 2:**
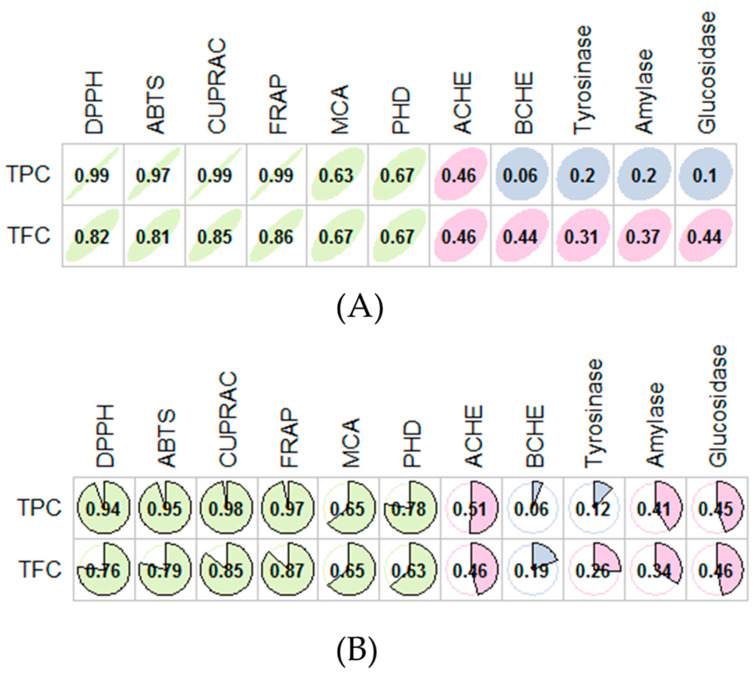
Relationship between the total phenolic content (TPC), total flavonoid content (TFC) and the biological activities. (**A**) Pearson correlation coefficient. (**B**) Spearman correlation coefficient.

In these assays, DPPH and ABTS were used to quench any free radicals and SCG extracts displayed higher radical quenching ability when compared with CS extracts. In both assays, the strongest radical scavenging ability was found in SCG EtOH:H_2_O extract, while the weakest ability was noted in water extract of CS samples. For reductive ability, CUPRAC and FRAP assays were performed which reflects the electron-donating ability of antioxidant compounds. Similar to DPPH and ABTS assays, the best activities were observed in SCG extracts and the highest one was again recorded from the SCG EtOH:H_2_O (501.85 mg TEg^−1^ for CUPRAC and 277.15 mg TE g^−1^ for FRAP). Altogether, the obtained results from radical scavenging and reducing power assays could be justified based on the higher concentration of phenolic compounds. This fact was confirmed by Pearson’s correlation analysis which showed a strong correlation between the total bioactive components and the radical scavenging and reducing power assays (Figure 2). In addition, the total amount of individual components in SCG extracts was higher than that of CS extracts. Our approach was also supported by some authors, who reported a positive relationship between the total bioactive components and the antioxidant properties [34,35,36].

In addition, the main compounds of coffee extracts, including caffeine and caffeoylquinic acid derivatives, have shown strong antioxidant activities in earlier reports [37,38,39,40,41]. For metal chelating ability, SCG MeOH:H_2_O extract showed the highest ability, followed by CS MeOH and SCG MeOH extracts. Similar to other assays, the CS H_2_O extract exerted the lowest chelating effect. This order of metal chelation differed from the radical scavenging and reducing power assays, and this observation could be linked to the non-phenolic chelator. This fact was also confirmed by the moderate Pearson correlation values for the metal chelation assay. In phosphomolybdenum assays, the ability of the tested samples decreased in the following order: SCG EtOH:H_2_O > CS MeOH > SCG MeOH:H_2_O > SCG MeOH > CS EtOH:H_2_O > SCG H_2_O > CS MEOH:H_2_O > CS H_2_O. Similar to the metal chelating assay, the phosphomolybdenum assay showed moderate correlation with total bioactive compounds. In the literature, the phosphomolybdenum assay is known as one of the total antioxidant assays and thus non-phenolic antioxidant compounds could contribute to the observed results [42,43,44].

### 3.3. Enzyme Inhibitory Effects

During the last century, the prevalence of some health problems is gaining much momentum worldwide. In this respect, lifestyle habits, imbalanced diet and stress are main reasons for increasing the prevalence of such diseases [45,46,47,48]. In this sense, according to some researchers, humanity can be considered to be in an ongoing war against these diseases, with an endeavor to find effective weapons to fight such challenges. In this context, several attempts to reduce morbidity and mortality associated with these diseases have been made from several fronts, yet with little success. Thus, researchers are seeking ways and means to reduce the prevalence of the above-mentioned diseases. Among the therapeutic approaches, one mechanism involves clinical enzymes linked to such pathologies, commonly known as enzyme inhibition theory [49]. Based on this theory, the inhibition of key metabolic enzymes might reduce the symptoms associated with these diseases. For example, cholinesterase hydrolyzes acetylcholine in synaptic cleavage. In Alzheimer’s patients, the level of acetylcholine is lower than that in a healthy person. In this sense, cholinesterase inhibition aims to retard degradation of acetylcholine and hence might improve cognitive functions associated with Alzheimer’s diseases [50]. Another example is diabetes mellitus, which is characterized by a high blood glucose level. Diabetes is responsible for symptoms observed, including neuropathy, cardiovascular and eye problems. Thus, the therapeutic strategy is geared towards lowering the blood glucose level and subsequently, alleviating these symptoms. Two enzymes, namely amylase and glucosidase, are the main enzymes in the hydrolysis of starch. The inhibition of amylase and glucosidase can retard the increase in the blood glucose level and hence be beneficial in the management of the glucose level after a carbohydrate-rich meal [51]. Altogether, these non-communicable diseases are treated with some synthetic enzyme inhibitors such as tacrine, donepezil (for Alzheimer’s disease), acarbose and miglitol (for diabetes mellitus). However, most of them have raised some concerns, thereby inducing side effects such as gastrointestinal disturbances and toxic properties [50,51,52]. Hence, there is still a dire need for the discovery of safe drugs in the treatment and/or management of the global health problems, especially from natural products such as medicinal and food plants.

In light of the aforementioned information, this study attempted to evaluate the inhibitory effects of by-product coffee extracts against a set of enzymes, including cholinesterases, amylase, glucosidase and tyrosinase. The results are summarized in Table 4.

For the AChE and BChE assays, the best ability was observed for the SCG MeOH extract. The water extracts from each sample exhibited the weakest inhibition on cholinesterases. As for the antidiabetic enzyme assays, EtOH:H_2_O and MeOH extracts had higher effects when compared with other extracts. Similar to cholinesterase, water extracts displayed the weakest ability. In contrast to the antidiabetic assays, MeOH and MeOH:H_2_O extracts were more active on tyrosinase, unlike the water extracts. As far as the literature could ascertain, compounds from coffee have been reported to be effective enzyme inhibitors. For example, caffeine, chlorogenic and caffeic acids exhibited promising enzyme inhibitory effects in in vitro and in vivo studies [18,19,20,21,53,54]. In addition, we observed a low correlation between the total bioactive compounds and the enzyme inhibitory effects. At this point, non-phenolic inhibitors such as alkaloids and terpenes might justify the activities observed. On another note, the observed results could be explained by the complex nature of these extracts and interactions (synergetic or antagonistic) between phytochemicals present therein.

### 3.4. Discriminant Analysis

CS and SCG are the most important by-products in the coffee industry. SCG is obtained during the brewing process with the hot water or steam, while CS is generated as a residue of the roasting process. These by-products have gained great interest as alternative cheapest sources to obtain added values of economic importance for the cosmetic, nutraceutical and pharmaceutical industries. In this connection, both coffee by-products seem to be a good source of bioactive compounds. However, among both by-products, we thought it useful to identify the most pharmaceutically efficient one. To this end, PLS-DA appeared to be the most appropriate statistical analysis technique. By using this supervised multivariate statistical technique, samples can be discriminated and the biological activities responsible for samples discrimination can be highlighted. In fact, PLS-DA is a robust regression technique widely used to perform classification models, since it enhances an optimal class separation and provides easily interpretable outcomes.

**Figure 3:**
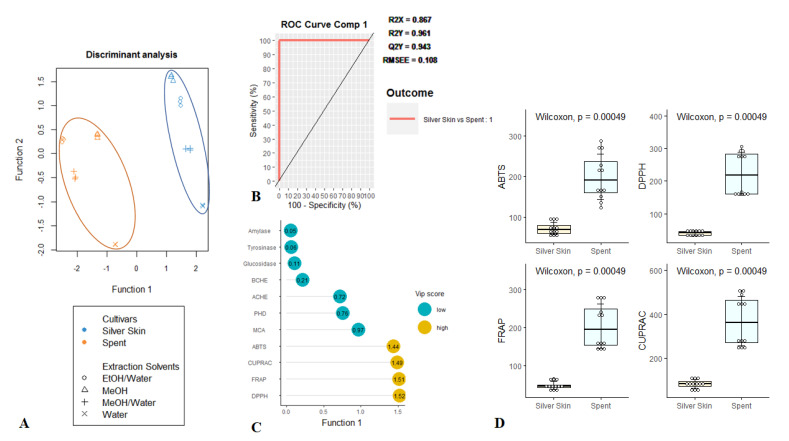
Supervised partial least squared discriminant analysis on the biological activities of coffee by-products. (**A**) Samples plot. (**B**) Model goodness parameters estimation, i.e., R^2^Y (goodness-of-fit), Q^2^Y (goodness-of-prediction), AUC (area under the curve average) and ROC (receiver operating characteristic) Curve using one-vs-all comparisons. (**C**) The most discriminant biological activities identifying though VIP (variable importance of projection) score calculation. (**D**) Characterization of coffee by-products taking account of the identified most discriminant biological activities.

Figure 3A depicting the results of statistical analysis, showed a separation of both coffee by-products along the first function of the model. The model was found to be successfully efficient to discriminate tested residues, recording high Q^2^Y, R^2^Y and AUC values, being 0.943, 0.961, and 1, respectively (Figure 3B). Afterwards, for the purpose of identifying the most discriminant assays accounting for the differences outlined in the model, the VIP (variable importance in projection) of each biological activity was estimated. Figure 3C shows the VIP scores obtained for the model, where CUPRAC, FRAP, DPPH and ABTS with values larger than 1 were found to be the most important assays for the two organs discrimination. A further investigation on these biological activities showed that SCG had higher antioxidant properties than CS (Figure 3D).

This is in agreement with the results obtained by Ballesteros et al. [55] and Conde and Mussatto [56], nevertheless the difference in the extraction techniques used in these studies. CS and SCG differ with regard to the antioxidant activities, although both have overall a relative similar enzyme inhibitor ability. This observation would result from the richness in caffeine, 5-CQA, 3-CQA and 3,5-diCQA, well-known as antioxidant compounds, in SCG extracts compared with those of CS. Especially, SCG EtOH:H_2_O contained the highest level of caffeine and this supported its higher antioxidant properties. This approach was also confirmed by PCA output (Figure 1) and the extract was noted as caffeine-rich. In addition, several researchers have reported that caffeine is a significant antioxidant. For example, Yashin et al. [41] reviewed the antioxidant properties of coffee and its components, and caffeine was found to exhibit strong antioxidant properties including free radical quenching and reductive abilities. In addition, one experimental study conducted by Azam et al. [37] indicated that caffeine had a protective action against DNA degradation by hydroxyl radicals. Demir et al. [57] reported that caffeine increased the renal antioxidant capacity in in vivo study. Thus, caffeine could be considered as a protective agent against oxidative stress. Similar positive effects for caffeine were also reported by Liu et al. [58] on the development of atherosclerosis in mice. Thus, SCG EtOH:H_2_O could be considered a valuable raw material with a high level of caffeine. Taken together, our findings suggest the possibility of reutilization of coffee by-products, in particular SCG, as natural antioxidant sources for application in pharmaceutical, nutraceutical and cosmeceutical products.

**Figure 4:**
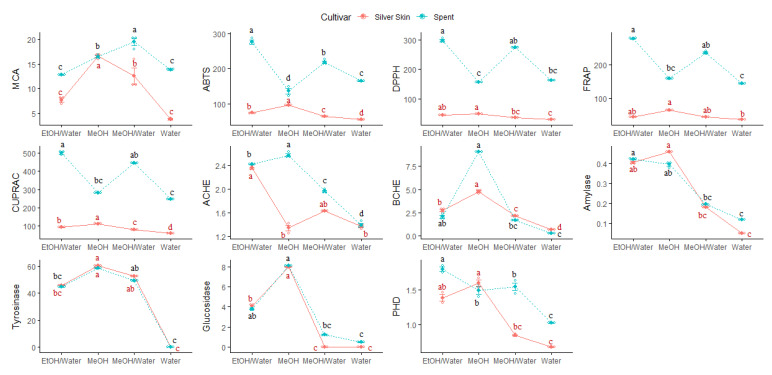
Dotplot for exploring the effect of extraction solvents on the biological activities of spent coffee ground and silverskin.

On the other hand, a high dispersion was observed in the distribution of the samples of the two respective parts, which is probably related to the different polarity of solvents used for extraction. Thus, as suggested by univariate analysis, a statistically significant difference was observed between the samples, for each individual coffee residue, which reflects the effect of the extraction solvent (Table S3). With regard to CS, the optimum biological activities, except for AChE, were obtained using MeOH as the extraction solvent (Figure 4).

Referring to our data on the phytochemical profiles, MeOH allowed to recover high contents of phenolics. Much more, among the phenolics, (-)-epicatechin was found just in the MeOH extract. Numerous studies concluded that (-)-epicatechin possesses high antioxidant activity and health effect in humans [59,60]. Concerning SCG samples, antioxidant properties and amylase inhibition were recorded in EtOH:H_2_O extract, the highest AChE, BChE, tyrosinase and glucosidase inhibition was found to be in the MeOH extract, whereas MeOH:H_2_O extract exerted the highest chelating ability (Figure 4). In fact, relative to the other extracts of SCG, EtOH:H_2_O extract was found to be rich in caffeic acid, syringic acid, *p*-coumaric acid, 3,5-diCQA and rutin, which can explain its high antioxidant activities.

SCG and CS contain considerable amounts of bioactive metabolites with remarkable biological activities. However, the difference in polarity of the solvent and the diverse chemical structure of the constituents lead to differences in the biological activities of each by-product-solvent. In fact, extraction is a crucial step in the recovery of molecules from these by-products. On the other hand, lower biological activities were remarkably observed in aqueous extracts from both samples. These findings may be due to the unipolar character of the plant cell walls, thereby allowing less polar solvents to extract the molecules from cells more effectively. Similar outcomes were obtained for mango and grape by-products, where less polar solvents proved to be more efficient for extraction of phytochemicals [61,62].

SCG extracts were proved to contain notable antioxidant and enzyme inhibitory molecules, thereby could be used as an inexpensive and natural alternative to synthetic food additives. However, it is well known that the employment of organic solvent in the food industry is strictly regulated. Thus, although for some biological activities, MeOH extracts showed remarkable properties, the condition of employment of MeOH is strict and limited. Accordingly, with regard to food security, in particular, it would be recommended to use EtOH:H_2_O solvent, showing excellent antioxidant activity, since it is better suited for employment in the manufacturing process of food ingredients.

## 4. Conclusions

This work provided an extensive characterization of the phytochemical profile of extracts obtained from two coffee by-products such as coffee silverskin and spent coffee ground. In addition, it reported a broad investigation on their antioxidant and enzyme inhibitory properties. The present research has contributed to increase the knowledge on coffee co-products in the hope that further innovative applications of coffee silverskin and spent coffee ground will be designed. In the perspective of more sustainable economy, the reuse of coffee by-products could lead to a drop in the waste released in the environment and the consequent cost of disposal. This may lead to an eco-friendlier coffee production and consumption.

## Figures and Tables

**Table 1:** Total bioactive components in the tested extracts ^1^.

	Extracts	Total Phenolic Content (mg GAE/g)	Total Flavonoid Content (mg RE/g)
SCG	EtOH:H_2_O (70:30)	93.55 ± 0.65 ^a^	4.37 ± 0.02 ^a^
	MeOH	63.25 ± 0.10 ^abc^	4.37 ± 0.16 ^a^
	MeOH:H_2_O (50:50)	93.26 ± 0.14 ^ab^	3.39 ± 0.27 ^ab^
	H_2_O	56.86 ± 0.16 ^abcd^	2.37 ± 0.10 ^abc^
CS	EtOH:H_2_O (70:30)	25.34 ± 0.44 ^cd^	0.18 ± 0.05 ^c^
	MeOH	35.68 ± 1.80 ^bcd^	2.32 ± 0.12 ^abc^
	MeOH:H_2_O (50:50)	25.02 ± 0.37 ^d^	0.86 ± 0.08 ^bc^
	H_2_O	20.49 ± 0.27 ^d^	0.75 ± 0.06 ^c^

^1^ Values expressed are means ± S.D. of three parallel measurements. GAE: Gallic acid equivalent; RE: Rutin equivalent. Different letters indicate significant differences in the tested extracts (*p* < 0.05).

**Table 2:** Contents (µg g^−1^ of dry weight extract) of bioactive compounds in SCG (spent coffee ground) and CS (coffee silverskin) extracts.

		Spent Coffee Ground Extracts (µg g^−1^)				Silverskin Extracts (µg g^−1^)			
No.	Analytes ^1^	MeOH	H_2_O	MeOH:H_2_O (50:50)	EtOH:H_2_O (70:30)	MeOH	H_2_O	MeOH:H_2_O (50:50)	EtOH:H_2_O (70:30)
1	Shikimic acid	n.d.^3^	n.d.	n.d.	n.d.	n.d.	n.d.	n.d.	n.d.
2	Gallic acid	42.45 ± 3.20	53.42 ± 4.10	51.90 ± 3.95	57.47 ± 5.03	32.65 ± 2.92	38.16 ± 1.58	43.25 ± 2.97	44.21 ± 3.45
3	5-CQA ^2^	9786.20 ± 815.36	4660.85 ± 254.32	10,613.61 ± 901.24	8620.83 ± 756.31	5436.78 ± 278.35	2589.36 ± 132.69	5896.45 ± 214.35	4789.35 ± 297.63
4	(+)-Catechin	1.23 ± 0.10	0.85 ± 0.06	1.56 ± 0.12	1.37 ± 0.10	n.d.	n.d.	n.d.	n.d.
5	Del-3,5-diglu ^2^	n.d.	n.d.	n.d.	n.d.	n.d.	n.d.	n.d.	n.d.
6	3-CQA ^2^	3377.82 ± 156.32	2091.89 ± 154.78	2736.86 ± 203.12	3777.65 ± 306.69	2598.32 ± 178.35	1549.55 ± 115.21	2657.15 ± 199.78	2698.32 ± 237.78
7	Caffeine	32,838.17 ± 2569.11	44,755.37 ± 3251.45	45,857.23 ± 4016.98	54,440.27 ± 4875.19	27,365.14 ± 1023.65	31,968.12 ± 1548.47	38,214.36 ± 1689.53	41,877.13 ± 2356.30
8	Cya-3-glu ^2^	1.56 ± 0.12	0.93 ± 0.08	1.65 ± 0.13	1.89 ± 0.15	n.d.	n.d.	n.d.	n.d.
9	Vanillic acid	86.61 ± 5.36	95.28 ± 8.21	140.38 ± 10.32	119.39 ± 9.96	96.23 ± 5.68	105.87 ± 9.23	116.98 ± 9.65	132.65 ± 10.65
10	Caffeic acid	67.98 ± 4.12	118.03 ± 8.65	156.60 ± 9.21	204.95 ± 15.45	45.32 ± 2.41	98.36 ± 5.68	142.36 ± 11.32	157.65 ± 10.56
11	(-)-Epicatechin	n.d.	n.d.	n.d.	n.d.	123.65 ± 10.63	n.d.	102.98 ± 8.65	n.d.
12	Syringic acid	33.65 ± 1.23	50.83 ± 4.56	50.26 ± 5.21	94.20 ± 8.32	n.d.	42.36 ± 2.63	45.69 ± 3.58	85.64 ± 6.82
13	*p*-Coumaric acid	7.10 ± 0.65	10.62 ± 1.03	13.30 ± 1.22	22.09 ± 1.98	5.46 ± 0.36	9.65 ± 0.79	10.23 ± 0.95	15.78 ± 1.03
14	Ferulic acid	55.36 ± 4.32	65.31 ± 5.36	125.36 ± 9.56	136.98 ± 11.54	38.69 ± 2.32	62.65 ± 4.01	97.69 ± 6.41	145.32 ± 10.85
15	3,5-diCQA ^2^	879.32 ± 65.32	852.36 ± 75.75	753.84 ± 54.21	1194.62 ± 98.96	115.63 ± 11.21	125.65 ± 8.95	358.97 ± 22.41	459.47 ± 49.8
16	Quinine	1.23 ± 0.09	1.56 ± 0.12	2.12 ± 0.19	3.65 ± 0.25	0.68 ± 0.02	0.71 ± 0.02	0.87 ± 0.03	0.93 ± 0.03
17	Naringin	0.36 ± 0.02	0.52 ± 0.04	0.31 ± 0.02	0.51 ± 0.03	n.d.	0.45 ± 0.03	n.d.	0.39 ± 0.02
18	Rutin	2.36 ± 0.15	3.54 ± 0.26	5.23 ± 0.47	9.65 ± 0.87	1.25 ± 0.06	2.65 ± 0.13	6.35 ± 0.54	10.65 ± 0.85
19	Hyperoside	0.65 ± 0.05	0.32 ± 0.02	0.54 ± 0.03	0.87 ± 0.06	0.56 ± 0.03	0.40 ± 0.02	0.59 ± 0.04	0.52 ± 0.02
20	*Trans*-cin acid ^2^	1.98 ± 0.12	2.35 ± 0.20	3.21 ± 0.31	4.95 ± 0.36	1.23 ± 0.04	2.56 ± 0.13	2.65 ± 0.14	4.87 ± 0.24
21	Resveratrol	n.d.	n.d.	n.d.	n.d.	n.d.	n.d.	n.d.	n.d.
22	Kae-3-glu ^2^	0.59 ± 0.03	0.47 ± 0.02	1.40 ± 0.09	1.89 ± 0.10	0.65 ± 0.04	0.36 ± 0.01	0.78 ± 0.05	1.26 ± 0.10
23	Quercitrin	0.43 ± 0.03	0.13 ± 0.01	0.62 ± 0.05	0.83 ± 0.07	0.36 ± 0.02	0.12 ± 0.01	0.41 ± 0.03	0.59 ± 0.05
24	Quercetin	3.74 ± 0.29	3.20 ± 0.28	3.76 ± 0.31	3.67 ± 0.33	3.12 ± 0.25	3.56 ± 0.25	3.42 ± 0.28	3.06 ± 0.20
25	Isogentisin	2.54 ± 0.19	1.05 ± 0.08	1.09 ± 0.09	0.99 ± 0.07	1.95 ± 0.15	0.95 ± 0.08	1.36 ± 0.11	1.10 ± 0.09
Total Compounds		47,191.31 ± 3058.21	52,768.88 ± 3658.54	60,520.82 ± 4536.41	68,698.72 ± 5012.36	35,867.67 ± 2898.32	36,601.49 ± 3025.45	47,702.54 ± 3987.36	50,428.89 ± 4023.74

^1^ Each sample was analyzed in triplicate (*n* = 3) and values are expressed as means ± S.D.; ^2^ 3-CQA, 3-Caffeoylquinic acid; 3,5-diCQA, 3,5-Dicaffeoylquinic acid; 5-CQA, 5-Caffeoylquinic acid; Del-3,5-diglu, Delphinidin-3,5-diglucosiede; Cya-3-glu, Cyanidin-3-glucoside; *Trans*-cin acid, *Trans*-cinnamic acid; Kae-3-glu, Kaempferol-3-glucoside; ^3^ n.d., not detectable.

**Table 3:** Antioxidant properties of the tested extracts ^1^.

	Extracts	DPPH (mg TE g^−1^)	ABTS (mg TE g^−1^)	CUPRAC (mg TE g^−1^)	FRAP (mg TE g^−1^)	Chel. ab. ^2^ (mg EDTAE g^−1^)	Pho. ^2^ (mmol TE g^−1^)
SCG	EtOH:H_2_O	296.78 ± 7.08 ^a^	276.19 ± 9.65 ^a^	501.85 ± 10.16 ^a^	277.15 ± 3.22 ^a^	12.82 ± 0.25 ^c^	1.80 ± 0.05 ^a^
	MeOH	156.13 ± 0.23 ^abcd^	136.11 ± 13.35 ^abcd^	280.96 ± 1.56 ^abc^	158.75 ± 0.90 ^ab^	16.45 ± 0.42 ^ab^	1.49 ± 0.09 ^abc^
	MeOH:H_2_O	274.44 ± 4.29 ^ab^	218.75 ± 6.88 ^ab^	446.39 ± 7.24 ^ab^	235.47 ± 4.98 ^ab^	19.47 ± 1.32 ^a^	1.54 ± 0.10 ^abc^
	H_2_O	163.72 ± 2.21 ^abc^	164.70 ± 1.55 ^abc^	246.90 ± 3.68 ^abcd^	143.61 ± 0.31 ^abc^	13.88 ± 0.26 ^bc^	1.03 ± 0.01 ^cd^
CS	EtOH:H_2_O	45.63 ± 0.11 ^cde^	73.66 ± 1.43 ^cde^	90.50 ± 0.78 ^cde^	45.76 ± 0.30 ^cd^	7.66 ± 0.71 ^d^	1.39 ± 0.08 ^bcd^
	MeOH	48.73 ± 0.09 ^bcde^	95.05 ± 0.04 ^bcde^	110.97 ± 0.87 ^bcde^	65.28 ± 1.91 ^bcd^	16.56 ± 0.45 ^ab^	1.60 ± 0.07 ^ab^
	MeOH:H_2_O	36.62 ± 0.58 ^de^	63.50 ± 1.65 ^de^	77.23 ± 1.24 ^de^	45.83 ± 0.19 ^bcd^	12.52 ± 2.96 ^c^	0.85 ± 0.02 ^cd^
	H_2_O	29.63 ± 0.14 ^e^	54.41 ± 0.76 ^e^	56.96 ± 0.74 ^e^	37.04 ± 0.20 ^d^	3.76 ± 0.31 ^e^	0.68 ± 0.01 ^d^

^1^ Values expressed are means ± S.D. of three parallel measurements. TE: Trolox equivalent; EDTAE: EDTA equivalent. Different letters indicate significant differences in the tested extracts (*p* < 0.05). ^2^ Chel. ab., Chelating ability; Pho., Phosphomolybdenum.

**Table 4:** Enzyme inhibitory properties of the tested extracts ^1^.

	Extracts	AChE Inhibition (mg GALAE g^−1^)	BChE Inhibition (mg GALAE g^−1^)	Tyrosinase Inhibition (mg KAE g^−1^)	Amylase Inhibition (mmol ACAE g^−1^)	Glucosidase Inhibition (mmol ACAE g^−1^)
SCG	EtOH:H_2_O	2.42 ± 0.01 ^a^	2.07 ± 0.27 ^abc^	44.73 ± 0.51 ^bcd^	0.42 ± 0.01 ^ab^	3.80 ± 0.18 ^abc^
	MeOH	2.57 ± 0.05 ^a^	9.07 ± 0.04 ^a^	58.42 ± 0.46 ^a^	0.40 ± 0.02 ^abc^	8.11 ± 0.03 ^a^
	MeOH:H_2_O	1.96 ± 0.03 ^ab^	1.73 ± 0.06 ^bc^	49.24 ± 0.71 ^abc^	0.20 ± 0.01 ^bcd^	1.21 ± 0.09 ^abcd^
	H_2_O	1.39 ± 0.06 ^bc^	0.29 ± 0.04 ^c^	Ni	0.12 ± 0.01 ^cd^	0.47 ± 0.05 ^bcd^
CS	EtOH:H_2_O	2.36 ± 0.03 ^a^	2.78 ± 0.22 ^ab^	45.84 ± 0.35 ^abcd^	0.41 ± 0.01 ^ab^	4.07 ± 0.28 ^ab^
	MeOH	1.35 ± 0.08 ^ce^	4.81 ± 0.19 ^a^	60.43 ± 0.18 ^a^	0.46 ± 0.01 ^a^	7.95 ± 0.05 ^a^
	MeOH:H_2_O	1.64 ± 0.01 ^abc^	2.19 ± 0.05 ^abc^	52.66 ± 0.31 ^ab^	0.18 ± 0.01 ^bcd^	Ni
	H_2_O	1.37 ± 0.04 ^bc^	0.72 ± 0.06 ^c^	Ni	0.05 ± 0.01 ^d^	Ni

^1^ Values expressed are means ± S.D. of three parallel measurements. GALAE: Galatamine equivalent; KAE: Kojic acid equivalent; ACAE: Acarbose equivalent. Ni: no inhibition. Different letters indicate significant differences in the tested extracts (*p* < 0.05).

## Supplementary Materials

The following are available online at https://www.mdpi.com/2304-8158/9/6/713/s1, Table S1. HPLC-MS/MS parameters in Dynamic-MRM mode including retention time (Rt) and delta retention time (Δ Rt) for each transition; Table S2: Result of normality test; Table S3: Normality checking and Univariate analysis for evaluating the effect of extraction solvents on biological activities of spent coffee ground and silverskin respectively.
