# Supplementary material for: Chemical Composition, Antioxidant and Enzyme Inhibitory Properties of Different Extracts Obtained from Spent Coffee Ground and Coffee Silverskin
Source: Foods. 2020 Jun 2;9(6):713. doi: 10.3390/foods9060713 (PMC7353581; doi:10.3390/foods9060713)
Supplement: Supplementary file 1 [file foods-09-00713-s001.pdf]

Chemical composition，antioxidant and enzyme inhibitory properties of different extracts obtained from spent coffee ground and coffee silverskin

Gokhan Zengin[1], Kouadio Ibrahime Sinan[1], Mohamad Fawzi Mahomoodally[2,3], Simone Angeloni[4,5], Ahmed M. Mustafa[4], Sauro Vittori[4], Filippo Maggi[4]* and Giovanni Caprioli[4]

1 Department of Biology, Science Faculty, Selcuk University, Campus, Konya, 42130 Konya, Turkey; gokhanzengin@selcuk.edu.tr (G.Z.) and sinankouadio@gmail.com (K.I.S.).
2 Institute of Research and Development, Duy Tan University, Da Nang 550000, Vietnam; mohamadfawzimahomoodally@duytan.edu.vn or f.mahomoodally@uom.ac.mu (M.F.M).
3 Department of Health Sciences, Faculty of Science, University of Mauritius, Réduit, Mauritius.
4 School of Pharmacy, University of Camerino, Via Sant'Agostino 1, 62032 Camerino, Italy. simone.angeloni@unicam.it (S.A.) ahmed.mustafa@unicam.it (A.M.), sauro.vittori@unicam.it (S.V.), filippo.maggi@unicam.it (F.M.) and giovanni.caprioli@unicam.it (G.C.).
5 International Hub for Coffee Research and Innovation, Via E. Betti 1, 62020 Belforte del Chienti, Italy.
* Correspondence: filippo.maggi@unicam.it; Tel.: +39-0737404506 (F.M.)

Assays for Total Phenolic and Flavonoid Contents

The total phenolic content was determined by employing the methods given in the literature with some modification. Sample solution (0.25 mL) was mixed with diluted Folin–Ciocalteu reagent (1 mL, 1:9, v/v) and shaken vigorously. After 3 min, Na_2CO_3 solution (0.75 mL, 1%) was added and the sample absorbance was read at 760 nm after a 2 h incubation at room temperature. The total phenolic content was expressed as milligrams of gallic acid equivalents (mg GAE/g extract)[1].

The total flavonoid content was determined using the $AlCl_3$ method. Briefly, sample solution (1 mL) was mixed with the same volume of aluminum trichloride (2%) in methanol. Similarly, a blank was prepared by adding sample solution (1 mL) to methanol (1 mL) without $AlCl_3$. The sample and blank absorbances were read at 415 nm after a 10 min incubation at room temperature. The absorbance of the blank was subtracted from that of the sample. Rutin was used as a reference standard and the total flavonoid content was expressed as milligrams of rutin equivalents (mg RE/g extract) [1].

Determination of Antioxidant and Enzyme Inhibitory Effects

Antioxidant (DPPH and ABTS radical scavenging, reducing power (CUPRAC and FRAP), phosphomolybdenum and metal chelating (ferrozine method)) and enzyme inhibitory activities (cholinesterase (Elmann's method), tyrosinase (dopachrome method), α-amylase (iodine/potassium iodide method), α -glucosidase (chromogenic PNPG method) and pancreatic lipase (*p*-nitrophenyl butyrate (p-NPB) method) were determined using the methods previously described by Uysal et al. [1] and Grochowski et al. [2]

For the DPPH (1,1-diphenyl-2-picrylhydrazyl) radical scavenging assay: Sample solution was added to 4 mL of a 0.004% methanol solution of DPPH. The sample absorbance was read at 517 nm after a 30 min incubation at room temperature in the dark. DPPH radical scavenging activity was expressed as milligrams of trolox equivalents (mg TE/g extract).

For ABTS (2,2′-azino-bis(3-ethylbenzothiazoline) 6-sulfonic acid) radical scavenging assay: Briefly, ABTS+ was produced directly by reacting 7 mM ABTS solution with 2.45 mM potassium persulfate and allowing the mixture to stand for 12–16 in the dark at room temperature. Prior to beginning the assay, ABTS solution was diluted with methanol to an absorbance of 0.700 ± 0.02 at 734 nm. Sample solution was added to ABTS solution (2 mL) and mixed. The sample absorbance was read at 734 nm after a 30 min incubation at room temperature. The ABTS radical scavenging activity was expressed as milligrams of trolox equivalents (mg TE/g extract).

For CUPRAC (cupric ion reducing activity) activity assay: Sample solution was added to premixed reaction mixture containing $CuCl_2$ (1 mL, 10 mM), neocuproine (1 mL, 7.5 mM) and NH_4Ac buffer (1 mL, 1 M, pH 7.0). Similarly, a blank was prepared by adding sample solution (0.5 mL) to premixed reaction mixture (3 mL) without $CuCl_2$. Then, the sample and blank absorbances

were read at 450 nm after a 30 min incubation at room temperature. The absorbance of the blank was subtracted from that of the sample. CUPRAC activity was expressed as milligrams of trolox equivalents (mg TE/g extract).

For FRAP (ferric reducing antioxidant power) activity assay: Sample solution was added to premixed FRAP reagent (2 mL) containing acetate buffer (0.3 M, pH 3.6), 2,4,6-tris(2-pyridyl)-S-triazine (TPTZ) (10 mM) in 40 mM HCl and ferric chloride (20 mM) in a ratio of 10:1:1 (v/v/v). Then, the sample absorbance was read at 593 nm after a 30 min incubation at room temperature. FRAP activity was expressed as milligrams of trolox equivalents (mg TE/g extract).

For phosphomolybdenum method: Sample solution was combined with 3 mL of reagent solution (0.6 M sulfuric acid, 28 mM sodium phosphate and 4 mM ammonium molybdate). The sample absorbance was read at 695 nm after a 90 min incubation at 95 °C. The total antioxidant capacity was expressed as millimoles of trolox equivalents (mmol TE/g extract).

For metal chelating activity assay: Briefly, sample solution was added to $FeCl_2$ solution (0.05 mL, 2 mM). The reaction was initiated by the addition of 5 mM ferrozine (0.2 mL). Similarly, a blank was prepared by adding sample solution (2 mL) to $FeCl_2$ solution (0.05 mL, 2 mM) and water (0.2 mL) without ferrozine. Then, the sample and blank absorbances were read at 562 nm after 10 min incubation at room temperature. The absorbance of the blank was sub-tracted from that of the sample. The metal chelating activity was expressed as milligrams of EDTA (disodium edetate) equivalents (mg EDTAE/g extract).

For Cholinesterase (ChE) inhibitory activity assay: Sample solution (was mixed with DTNB (5,5-dithio-bis(2-nitrobenzoic) acid, Sigma, St. Louis, MO, USA) (125 μL) and AChE (acetylcholines-terase (Electric ell acetylcholinesterase, Type-VI-S, EC 3.1.1.7,Sigma)), or BChE (butyrylcholinesterase (horse serum butyrylcholinesterase, EC 3.1.1.8, Sigma)) solution (25 μL) in

Tris–HCl buffer (pH 8.0) in a 96-well microplate and incubated for 15 min at 25 °C. The reaction was then initiated with the addition of acetylthiocholine iodide (ATCI, Sigma) or butyrylthiocholine chloride (BTCl, Sigma) (25 μL). Similarly, a blank was prepared by adding sample solution to all reaction reagents without enzyme (AChE or BChE) solution. The sample and blank absorbances were read at 405 nm after 10 min incubation at 25 °C. The absorbance of the blank was subtracted from that of the sample and the cholinesterase inhibitory activity was expressed as galanthamine equivalents (mgGALAE/g extract).

For Tyrosinase inhibitory activity assay: Sample solution was mixed with tyrosinase solution (40 μL, Sigma) and phosphate buffer (100 μL, pH 6.8) in a 96-well microplate and incubated for 15 min at 25 °C. The reaction was then initiated with the addition of L-DOPA (40 μL, Sigma). Similarly, a blank was prepared by adding sample solution to all reaction reagents without enzyme (tyrosinase) solution. The sample and blank absorbances were read at 492 nm after a 10 min incubation at 25 °C. The absorbance of the blank was subtracted from that of the sample and the tyrosinase inhibitory activity was expressed as kojic acid equivalents (mgKAE/g extract).

For α-amylase inhibitory activity assay: Sample solution was mixed with α-amylase solution (ex-porcine pancreas, EC 3.2.1.1, Sigma) (50 μL) in phosphate buffer (pH 6.9 with 6 mM sodium chloride) in a 96-well microplate and incubated for 10 min at 37 °C. After pre-incubation, the reaction was initiated with the addition of starch solution (50 μL, 0.05%). Similarly, a blank was prepared by adding sample solution to all reaction reagents without enzyme (α-amylase) solution. The reaction mixture was incubated 10 min at 37 °C. The reaction was then stopped with the addition of HCl (25 μL, 1 M). This was followed by addition of the iodine-potassium iodide solution (100 μL). The sample and blank absorbances were read at 630 nm. The absorbance of the blank

was subtracted from that of the sample and the α-amylase inhibitory activity was expressed as acarbose equivalents (mmol ACE/g extract).

For α-glucosidase inhibitory activity assay: Sample solution was mixed with glutathione (50 μL), α-glucosidase solution (from Saccharomyces cerevisiae, EC 3.2.1.20, Sigma) (50 μL) in phosphate buffer (pH 6.8) and PNPG (4-N-trophenyl-α-D-glucopyranoside, Sigma) (50 μL) in a 96-well microplate and incubated for 15 min at 37 °C. Similarly, a blank was prepared by adding sample solution to all reaction reagents without enzyme (α-glucosidase) solution. The reaction was then stopped with the addition of sodium carbonate (50 μL, 0.2 M). The sample and blank absorbances were read at 400 nm. The absorbance of the blank was subtracted from that of the sample and the α-glucosidase inhibitory activity was expressed as acarbose equivalents (mmol ACE/g extract).

Table S1. Supplementary materials. HPLC-MS/MS parameters in Dynamic-MRM mode including retention time (Rt) and delta retention time (Δ Rt) for each transition.

No.	Compounds	Precursor ion (*m/z*)	Product ion (*m/z*)	Fragmentor (V)	Collision energy (V)	Polarity	Retention time (min)	Delta retention time (Δ Rt)
1	Shikimic acid	173	173<br>-	87<br>-	0<br>-	Negative<br>-	1.40	3
2	Gallic acid	169	125[a]<br>51	92	12<br>36	Negative	2.37	3
3	5-Caffeoylquinic acid	353	191[a]<br>179	102	12<br>12	Negative	3.58	3
4	(+)-Catechin	289	245[a]<br>109	121	8<br>24	Negative	5.48	3
5	Delphinidin-3,5-diglucoside	463	300[a]<br>271	165	24<br>48	Negative	5.64	3
6	3-Caffeoylquinic acid	353	191[a]<br>85	92	12<br>48	Negative	6.22	3
7	Caffeine	195	138[a]<br>110	107	20<br>24	Positive	6.50	3
8	Cyanidin-3-glucoside	449	287[a]<br>403	121	20<br>16	Positive	6.50	3
9	Vanillic acid	167	108[a]<br>152	78	16<br>8	Negative	6.70	3
10	Caffeic acid	179	135[a]<br>134	87	12<br>24	Negative	6.87	3
11	(-)-Epicatechin	289	245[a]<br>109	126	8<br>20	Negative	7.03	3
12	Syringic acid	197	182[a]<br>123	92	8<br>20	Negative	7.48	3
13	*p*-Coumaric acid	163	119[a]<br>93	83	12<br>32	Negative	8.47	3
14	Ferulic acid	193	134[a]<br>178	88	12<br>8	Negative	9.16	3
15	3,5-Dicaffeoylquinic acid	515	353[a]<br>191	117	8<br>28	Negative	9.82	3
16	Quinine	325	79[a]<br>81	135	44<br>32	Positive	10.1	5
17	Naringin	579	271[a]<br>151	210	32<br>48	Negative	10.17	3
18	Rutin	609	300[a]<br>271	195	40<br>50	Negative	10.34	3

19	Hyperoside	463	300[a]<br>271	160	24<br>44	Negative	10.43	3
20	*Trans*-cinnamic acid	149	131[a]<br>77	44	8<br>36	Positive	10.79	3
21	Resveratrol	227	185[a]<br>143	131	12<br>20	Negative	10.92	3
22	Kaempferol-3-glucoside	447	284[a]<br>227	163	24<br>50	Negative	11.24	3
23	Quercitrin	447	300[a]<br>301	155	24<br>16	Negative	11.24	3
24	Quercetin	301	151[a]<br>179	126	16<br>12	Negative	13.03	3
25	Isogentisin	257	242[a]<br>214	116	16<br>24	Negative	16.31	3

[a] These product ions were used for quantification, the others to confirm the analytes.

Table S2. Result of normality test.

Biological activities	Shapiro-wilk	Anderson-darling
TPC	0,001	0,001
TFC	0,013	0,020
DPPH	0,000	< 0,0001
ABTS	0,008	0,010
CUPRAC	0,001	0,001
FRAP	0,001	0,001
MCA	0,101	0,103
PHD	0,039	0,031
ACHE	0,004	0,002
BCHE	0,000	< 0,0001
Tyrosinase	< 0,0001	< 0,0001
Amylase	0,002	0,000
Glucosidase	0,000	0,000

Values expressed are *p*-value. Variable with $p > 0.05$ is normal distributed

Table S3. Normality checking and Univariate analysis for evaluating the effect of extraction solvents on biological activities of Spent and Silver skin respectively.

Biological activities	Spent				Silver skin			
	Normality tests		Kruskal-Wallis	One-way ANOVA	Normality tests		Kruskal-Wallis	One-way ANOVA
	Shapiro-wilk	Anderson-Darling			Shapiro-wilk	Anderson-Darling		
DPPH	0,003	0,001	0,016		0,031	0,039	0,016	
ABTS	0,281	0,282		< 0,0001	0,053	0,082		< 0,0001
CUPRAC	0,012	0,008	0,016		0,160	0,255		< 0,0001
FRAP	0,016	0,015	0,016		0,010	0,004	0,025	
MCA	0,144	0,199		< 0,0001	0,091	0,147		< 0,0001
PHD	0,123	0,161		< 0,0001	0,043	0,041	0,016	
ACHE	0,053	0,061		< 0,0001	0,006	0,003	0,025	
BCHE	0,001	0,000	0,015		0,133	0,175		< 0,0001
Tyrosinase	0,002	0,000	0,015		0,002	0,000	0,015	
Amylase	0,007	0,005	0,016		0,016	0,017	0,016	
Glucosidase	0,011	0,012	0,016		0,004	0,003	0,014	

Values expressed are *p*-value. Shapiro-wilk & Anderson-Darling: Variable with $p > 0.05$ follows a Normal distribution. Kruskal-Wallis & One-way ANOVA: Statistically significant difference was observed ($p < 0.05$)

References

1. Uysal, S.; Zengin, G.; Locatelli, M.; Bahadori, M. B.; Mocan, A.; Bellagamba, G.; De Luca, E.; Mollica, A.; Aktumsek, A., Cytotoxic and enzyme inhibitory potential of two Potentilla species (P. speciosa L. and P. reptans Willd.) and their chemical composition. *Frontiers in pharmacology* **2017,** 8, 290.
2. Grochowski, D. M.; Uysal, S.; Aktumsek, A.; Granica, S.; Zengin, G.; Ceylan, R.; Locatelli, M.; Tomczyk, M., In vitro enzyme inhibitory properties, antioxidant activities, and phytochemical profile of Potentilla thuringiaca. *Phytochemistry Letters* **2017,** 20, 365-372.
